# Genome sequence of the exopolysaccharide-producing *Salipiger mucosus* type strain (DSM 16094^T^), a moderately halophilic member of the *Roseobacter* clade

**DOI:** 10.4056/sigs.4909790

**Published:** 2014-03-15

**Authors:** Thomas Riedel, Stefan Spring, Anne Fiebig, Jörn Petersen, Nikos C. Kyrpides, Markus Göker, Hans-Peter Klenk

**Affiliations:** 1 Sorbonne Universités, UPMC Univ Paris 06, USR 3579, LBBM, Observatoire Océanologique, Banyuls/Mer, France; 2 CNRS, USR 3579, LBBM, Observatoire Océanologique, Banyuls/Mer, France; 3 Leibniz Institute DSMZ – German Collection of Microorganisms and Cell Cultures, Braunschweig, Germany; 4 DOE Joint Genome Institute, Walnut Creek, California, USA

**Keywords:** aerobic, chemoheterotrophic, rod-shaped, photosynthesis, extrachromosomal elements, OmniLog phenotyping, *Roseobacter* clade, *Rhodobacteraceae*, *Alphaproteobacteria*

## Abstract

*Salipiger mucosus* Martínez-Cànovas *et al.* 2004 is the type species of the genus *Salipiger*, a moderately halophilic and exopolysaccharide-producing representative of the *Roseobacter* lineage within the alphaproteobacterial family *Rhodobacteraceae*. Members of this family were shown to be the most abundant bacteria especially in coastal and polar waters, but were also found in microbial mats and sediments. Here we describe the features of the *S. mucosus* strain DSM 16094^T^ together with its genome sequence and annotation. The 5,689,389-bp genome sequence consists of one chromosome and several extrachromosomal elements. It contains 5,650 protein-coding genes and 95 RNA genes. The genome of *S. mucosus* DSM 16094^T^ was sequenced as part of the activities of the Transregional Collaborative Research Center 51 (TRR51) funded by the German Research Foundation (DFG).

## Introduction

The *Roseobacter* clade is a very heterogeneous group of marine *Alphaproteobacteria* that plays an important role in the global carbon cycle and other biogeochemical processes [1]. Members of this group form an allegedly monophyletic, physiologically heterogeneous, as well as metabolically versatile group of bacterioplankton [1]. They are known to live in the open ocean, especially in coastal areas, where they have been found many times in symbiosis with algae, in microbial mats, sediments, or associated with invertebrates, but representatives of this lineage were also isolated from marine environments like polar waters or sea ice [1-4], which is also presented and reflected by their genome sequences [2]. Whereas some members of the *Roseobacter* clade contain the pigment bacteriochlorophyll *a* and are capable of aerobic anoxygenic photophosphorylation, other members were found to transform dimethylsulfonylpropionate into dimethylsulfide [4-6].

Some representatives of the *Roseobacter* lineage such as *Salipiger mucosus* A3^T^ are also known to be moderate halophiles, which are adapted to a wide range of salinities and were found to produce special compounds like compatible solutes, halophilic enzymes or exopolysaccharides [7-9].

Strain A3^T^ (= DSM 16094^T^ = LMG 22090^T^ = CECT 5855^T^) represents the type strain of *S. mucosus* (initially proposed as ‘*S. muscescens’*) in the monotypic genus *Salipiger* [10] and was isolated from saline soil bordering a saltern on the Mediterranean Sea coast at Calblanque (Spain) [7]. The genus name *Salipiger* was derived from the Latin noun *sal, salis* (‘salt’) and the Latin adjective *piger* (‘lazy’) [10]. The species epithet *mucosus* refers to the Latin adjective *mucosus* (‘slimy, mucous’) [10]. Current PubMed records do not indicate any follow-up research with strain A3^T^ after the initial description of *S. mucosus* [7] and the characterization of its exopolysaccharide [11].

In this study we analyzed the genome sequence of *S. mucosus* DSM 16094^T^, which was selected for sequencing under the auspices of the German Research Foundation (DFG) Transregio-SFB51 *Roseobacter* grant because of its phylogenetic position [12] and was also a candidate for the Genomic Encyclopedia of *Archaea* and *Bacteria* [13]. We present a description of the genomic sequencing and annotation and present a summary classification together with a set of features for strain DSM 16094^T^, including novel aspects of its phenotype.

## Classification and features

### 16S rRNA gene analysis

The single genomic 16S rRNA gene sequence of *S. mucosus* DSM 16094^T^ was compared with the Greengenes database for determining the weighted relative frequencies of taxa and (truncated) keywords as previously described [14]. The most frequently occurring genera were *Salipiger* (21.2%), *Pelagibaca* (17.1%), *Roseovarius* (17.0%), *Marinovum* (13.1%) and *Roseobacter* (9.5%) (30 hits in total). Regarding the single hit to sequences from members of the species, the average identity within high scoring pairs (HSPs) was 100.0%, whereas the average coverage by HSPs was 97.2%. Regarding the two hits to sequences from other members of the genus, the average identity within HSPs was 98.4%, whereas the average coverage by HSPs was 99.0%. Among all other species, the one yielding the highest score was *'Salipiger bermudensis'* (DQ178660), which corresponded to an identity of 96.8% and a HSP coverage of 99.9%. (Note that the Greengenes database uses the INSDC (= EMBL/NCBI/DDBJ) annotation, which is not an authoritative source for nomenclature or classification). The highest-scoring environmental sequence was AB302369 (Greengenes short name 'Hydrocarbon-Degrading Indonesian Seawater seawater isolate B44-2B44-2 str. B44-2'), which showed an identity of 97.4% and an HSP coverage of 95.4%. The most frequently occurring keywords within the labels of all environmental samples that yielded hits were 'aquat, rank' (4.9%), 'microbi' (3.7%), 'harbour, newport' (3.3%), 'water' (2.7%) and 'seawat' (2.4%) (219 hits in total) and in line with the habitat from which strain A3^T^ was isolated. Environmental samples that yielded hits of a higher score than the highest scoring species were not found.

**Figure 1:**
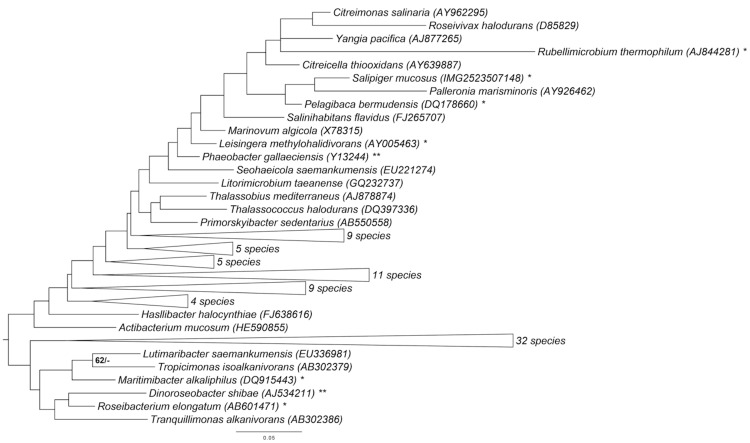
Phylogenetic tree highlighting the position of *S. mucosus* relative to the type strains of the type species of the other genera within the family *Rhodobacteraceae*. The tree was inferred from 1,331 aligned characters of the 16S rRNA gene sequence under the maximum likelihood (ML) criterion as previously described [14]. Rooting was done initially using the midpoint method [15] and then checked for its agreement with the current classification (Table 1). The branches are scaled in terms of the expected number of substitutions per site. Numbers adjacent to the branches are support values from 650 ML bootstrap replicates (left) and from 1,000 maximum-parsimony bootstrap replicates (right) if larger than 60% [14]. (That is, the backbone of the tree is largely unresolved.) Lineages with type strain genome sequencing projects registered in GOLD [16] are labeled with one asterisk, those also listed as 'Complete and Published' with two asterisks [3,17].

Figure 1 shows the phylogenetic neighborhood of *S. mucosus* strain DSM 16094^T^ in a 16S rRNA gene sequence based tree. The sequence of the single 16S rRNA gene copy in the genome does not differ from the previously published 16S rRNA gene sequence (AY527274), which contains two ambiguous base calls.

**Table 1:** Classification and general features of *S. mucosus* DSM 16094^T^ according to the MIGS recommendations [18] (published by the Genome Standards Consortium [19]).

**MIGS ID**	**Property**	**Term**	**Evidence code**
		Domain *Bacteria*	TAS [20]
		Phylum *Proteobacteria*	TAS [21]
		Class *Alphaproteobacteria*	TAS [22,23]
	Current classification	Order *Rhodobacterales*	TAS [23,24]
		Family *Rhodobacteraceae*	TAS [25]
		Genus *Salipiger*	TAS [7]
		Species *Salipiger mucosus*	TAS [7,26]
		Strain A3^T^	TAS [1]
	Gram stain	negative	TAS [1]
	Cell shape	rod-shaped	TAS [1]
	Motility	non-motile	TAS [1]
	Sporulation	not reported	
	Temperature range	20-40°C	TAS [1]
	Optimum temperature	30°C	NAS
	Salinity	0.5-20% (Sea Salts)	TAS [1]
MIGS-22	Oxygen requirement	strictly aerobic	TAS [1]
	Carbon source	complex (e.g., yeast extract, peptone)_	TAS [1]
	Energy metabolism	chemoheterotroph	TAS [1]
MIGS-6	Habitat	hypersaline soil	TAS [1]
MIGS-15	Biotic relationship	free living	TAS [1]
MIGS-14	Pathogenicity	none	NAS
	Biosafety level	1	TAS [27]
MIGS-23.1	Isolation	hypersaline soil bordering a saltern	TAS [1]
MIGS-4	Geographic location	Calblanque, Murcia (southeastern Spain)	TAS [1]
MIGS-5	Sample collection time	1998	NAS
MIGS-4.1	Latitude	37.64	NAS
MIGS-4.2	Longitude	-0.77	NAS_
MIGS-4.3	Depth	not reported	
MIGS-4.4	Altitude	not reported	

Evidence codes - TAS: Traceable Author Statement (i.e., a direct report exists in the literature); NAS: Non-traceable Author Statement (i.e., not directly observed for the living, isolated sample, but based on a generally accepted property for the species, or anecdotal evidence). Evidence codes are from of the Gene Ontology project [28].

### Morphology and physiology

**Figure 2:**
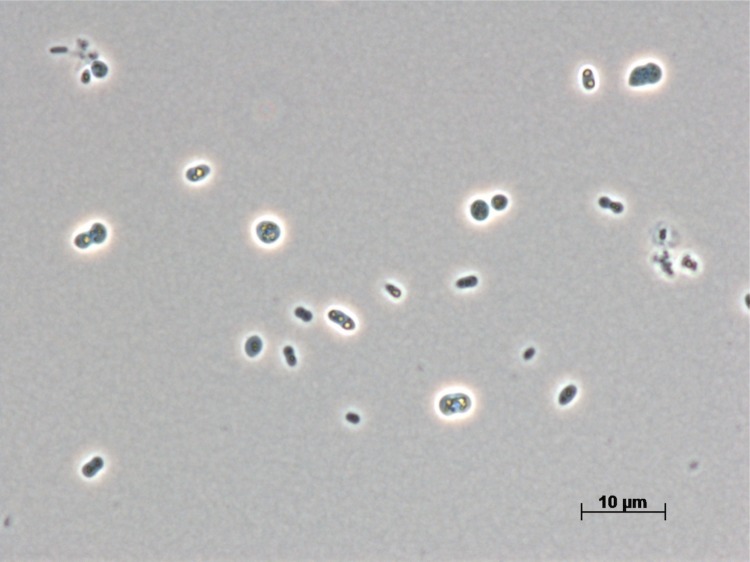
Phase-contrast micrograph of *S. mucosus* DSM 16094^T^.

Cells of strain A3^T^ are pleomorphic and stain Gram-negative (Figure 2). They are 1 µm in width and 2.0-2.5 µm in length. Motility was not observed. They live a strictly aerobic and chemoheterotrophic lifestyle. Colonies grown on MY solid medium are circular, convex, cream-colored and mucoid, whereas in liquid medium their growth is uniform. Cells are encapsulated. They are moderately halophilic, and capable of growth in a mixture of sea salts from 0.5 to 20% (w/v), whereas the optimum is between 3 and 6% (w/v) sea salts. When using NaCl instead of sea salts, optimal growth occurs at a salt concentration of 9-10% (w/v). Cells of strain A3^T^ grow within a temperature range of 20-40°C and at a pH range between 6 and 10. Growth does not occur under anaerobic conditions either by fermentation, fumarate or nitrate reduction or photoheterotrophy. Cells are cytochrome oxidase and catalase positive. Polyhydroxyalkanoates (PHA) are stored as reserve material within the cells. H_2_S is produced from L-cysteine. Selenite reduction, gluconate oxidation and phosphatase were observed. Urea and Tween 20 are hydrolyzed. A variety of tested organic compounds were neither metabolized nor sustained growth; for details see [7].

The utilization of carbon compounds by *S. mucosus* DSM 16094^T^ grown at 28°C was also determined for this study using Generation-III microplates in an OmniLog phenotyping device (BIOLOG Inc., Hayward, CA, USA). The microplates were inoculated with a cell suspension at a cell density of 95-96% turbidity and dye IF-A. Further additives were vitamins, micronutrient and sea-salt solutions, which had to be added for dealing with such marine bacteria [29]. The plates were sealed with parafilm to avoid a loss of fluid. The exported measurement data were further analyzed with the opm package for R [30,31], using its functionality for statistically estimating parameters from the respiration curves such as the maximum height, and automatically translating these values into negative, ambiguous, and positive reactions. The reactions were recorded in three individual biological replicates.

The strain was positive for pH 6, 1% NaCl, 4% NaCl, 8% NaCl, D-galactose, 3-O-methyl-D-glucose, D-fucose, L-fucose, L-rhamnose, 1% sodium lactate, *myo*-inositol, rifamycin SV, L-aspartic acid, L-glutamic acid, L-histidine, L-serine, D-glucuronic acid, quinic acid, L-lactic acid, citric acid, *α*-keto-glutaric acid, D-malic acid, L-malic acid, nalidixic acid, sodium formate and the positive control.

No reactions could be detected for the negative control, dextrin, D-maltose, D-trehalose, D-cellobiose, *β*-gentiobiose, sucrose, D-turanose, stachyose, pH 5, D-raffinose, *α*-D-lactose, D-melibiose, *β*-methyl-D-galactoside, D-salicin, *N*-acetyl-D-glucosamine, *N*-acetyl-*β*-D-mannosamine, *N*-acetyl-D-galactosamine, *N*-acetyl-neuraminic acid, D-glucose, D-mannose, D-fructose, inosine, fusidic acid, D-serine, D-sorbitol, D-mannitol, D-arabitol, glycerol, D-glucose-6-phosphate, D-fructose-6-phosphate, D-aspartic acid, D-serine, troleandomycin, minocycline, gelatin, glycyl-L-proline, L-alanine, L-arginine, L-pyroglutamic acid, lincomycin, guanidine hydrochloride, niaproof, pectin, D-galacturonic acid, L-galactonic acid-*γ*-lactone, D-gluconic acid, glucuronamide, mucic acid, D-saccharic acid, vancomycin, tetrazolium violet, tetrazolium blue, p-hydroxy-phenylacetic acid, methyl pyruvate, D-lactic acid methyl ester, bromo-succinic acid, lithium chloride, potassium tellurite, tween 40, *γ*-amino-n-butyric acid, *γ*-hydroxy-butyric acid, *β*-hydroxy-butyric acid, *α*-keto-butyric acid, acetoacetic acid, propionic acid, acetic acid, aztreonam, butyric acid and sodium bromate.

Martínez-Cánovas *et al.* tested the strain A3^T^ for growth on a variety of substrates, none of which were utilized under the applied conditions [7]. In contrast to this result, OmniLog measurements detected respiration in nearly twenty wells, including several sugars and amino acids. This may be due to respiratory measurements being more sensitive than growth measurements [32]. For instance, the positive reactions detected only in the OmniLog instrument might be caused by substrates that were only partially metabolized.

An important physiological property, the halophilic lifestyle, could be confirmed by the OmniLog measurements, showing that *S. mucosus* is able to grow in up to 8% NaCl. According to [7] the salt tolerance of this strain exceeds 10% NaCl.

### Chemotaxonomy

The principal cellular fatty acids of strain A3^T^ are C_18:1 ω7c_ (78.0%), C_16:0_ (12.4%), C_12:1 3-OH_ (2.3%), C_19:0 cyclo ω8c_ (2.3%), C_18:0_ (2.0%) and C_16:1 ω7c_ and/or C_15:0_ *_iso_*_2-OH_ (1.3%). The presence of C_18:1 ω7c_ as predominant fatty acid is a feature characteristic of several taxa within the *Alphaproteobacteria* (e.g. *Jannaschia helgolandensis* DSM 14858^T^ [33], *Octadecabacter arcticus* CIP 106731^T^ [34], *Ruegeria algicola* ATCC 51440^T^ [35], *Sulfitobacter mediterraneus* DSM 12244^T^ [36] and *Staleya guttiformis* DSM 14443^T^ [37]).

The only detected respiratory lipoquinone was ubiquinone 10, which is a well-known characteristic of alphaproteobacterial representatives (all data from [7]).

## Genome sequencing and annotation

### Genome project history

The genome of *S. mucosus* DSM 16094^T^ was sequenced as a part of the DFG funded project TRR51 “Ecology, Physiology and Molecular Biology of the *Roseobacter* clade: Towards a Systems Biology Understanding of a Globally Important Clade of Marine Bacteria”. The strain was chosen for genome sequencing according the Genomic Encyclopedia of Bacteria and Archaea (GEBA) criteria [12,13]. Project information can found in the Genomes OnLine Database [16]. The genome sequence is deposited in GenBank and the Integrated Microbial Genomes database (IMG) [38]. A summary of the project information is shown in Table 2.

**Table 2:** Genome sequencing project information

MIGS ID	Property	Term
MIGS-31	Finishing quality	Non-contiguous finished
MIGS-28	Libraries used	Two genomic libraries: one Illumina PE library (420 bp insert size), one 454 PE library (3kb insert size
MIGS-29	Sequencing platforms	Illumina GA IIx, Illumina MiSeq, 454 GS-FLX+ Titanium
MIGS-31.2	Sequencing coverage	430×
MIGS-30	Assemblers	velvet version 1.1.36, Newbler version 2.3, consed 20.0
MIGS-32	Gene calling method	Prodigal 1.4
	INSDC ID	APVH00000000
	GenBank Date of Release	July 31th, 2013
	GOLD ID	Gi0042373
	NCBI project ID	188761
	Database: IMG	2523231081
MIGS-13	Source material identifier	DSM 16094
	Project relevance	Tree of Life, biodiversity

### Growth conditions and DNA isolation

A culture of *S. mucosus* DSM 16094^T^ was grown aerobically in DSMZ medium 512 [39] by adding 2.5% NaCl at a temperature of 30°C. Genomic DNA was isolated using Jetflex Genomic DNA Purification Kit (GENOMED 600100) following the standard protocol provided by the manufacturer but modified by an incubation time of 60 min, the incubation on ice overnight on a shaker, the use of additional 50 μl proteinase K, and the addition of 100 μl protein precipitation buffer. The DNA is available from the Leibniz-Institute DSMZ through the DNA Bank Network [40].

### Genome sequencing and assembly

The genome was sequenced using a combination of two genomic libraries (Table 2). Sequencing and assembly were performed according to the protocol established for the genome of *R. thermophilum* DSM 16684^T^ [17] with the following additional step. To achieve longer reads, the Illumina library was sequenced in one direction for 300 cycles, providing another 15.0 million reads. The hybrid assembly consisted of 14,800,324 filtered Illumina reads with a median length of 201 bp. Pyrosequencing resulted in 53,566 reads with an average read length of 308 bp. After manual editing, the final assembly was composed of 84 contigs organized in 30 scaffolds. The combined sequences provided a 430× genome coverage.

### Genome annotation

Genome annotation was carried out using the JGI genome annotation pipeline as previously described [17].

## Genome properties

**Figure 3:**
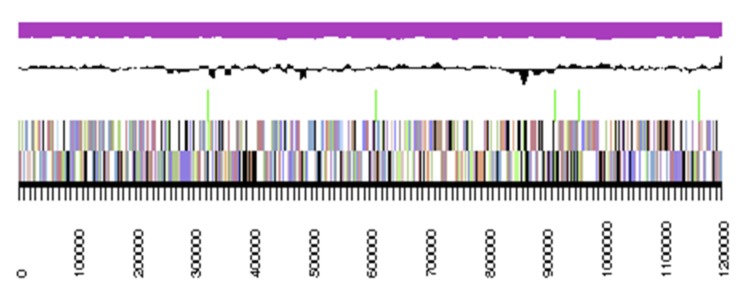
Graphical map of the largest scaffold. From bottom to the top: Genes on forward strand (colored by COG categories), Genes on reverse strand (colored by COG categories), RNA genes (tRNAs green, rRNAs red), GC content (black), GC skew (purple/olive).

The genome statistics are provided in Table 3 and Figure 3. The genome of strain DSM 16094^T^ has a total length of 5,689,389 bp and a G+C content of 67.1%. Of the 5,745 genes predicted, 5,650 were protein-coding genes, and 95 RNAs. The majority of the protein-coding genes (76.0%) were assigned a putative function while the remaining ones were annotated as hypothetical proteins. The distribution of genes into COGs functional categories is presented in Table 4.

**Table 3:** Genome Statistics

**Attribute**	Value	% of Total
Genome size (bp)	5,689,389	100.00
DNA coding region (bp)	5,064,332	89.01
DNA G+C content (bp)	3,815,108	67.06
Number of scaffolds	30	
Total genes	5,745	100.00
RNA genes	95	1.65
rRNA operons	4	
tRNA genes	83	1.44
Protein-coding genes	5,650	98.35
Genes with function prediction (proteins)	4,365	75.98
Genes in paralog clusters	4,666	81.22
Genes assigned to COGs	4,191	72.95
Genes assigned Pfam domains	4,465	77.91_
Genes with signal peptides	512	8.91
Genes with transmembrane helices	1,183	20.59
CRISPR repeats	1	

**Table 4:** Number of genes associated with the general COG functional categories

**Code**	**Value**	**%age**	**Description**
J	175	3.8	Translation, ribosomal structure and biogenesis
A	0	0.0	RNA processing and modification
K	350	7.5	Transcription
L	228	4.9	Replication, recombination and repair
B	4	0.1	Chromatin structure and dynamics
D	37	0.8	Cell cycle control, cell division, chromosome partitioning
Y	0	0.0	Nuclear structure
V	47	1.0	Defense mechanisms
T	179	3.8	Signal transduction mechanisms
M	289	6.2	Cell wall/membrane/envelope biogenesis
N	96	2.1	Cell motility
Z	1	0.0	Cytoskeleton
W	0	0.0	Extracellular structures
U	84	1.8	Intracellular trafficking and secretion, and vesicular transport
O	141	3.0	Posttranslational modification, protein turnover, chaperones
C	289	6.2	Energy production and conversion
G	362	7.8	Carbohydrate transport and metabolism
E	507	10.9	Amino acid transport and metabolism
F	95	2.0	Nucleotide transport and metabolism
H	174	3.7	Coenzyme transport and metabolism
I	172	3.7	Lipid transport and metabolism
P	253	5.4	Inorganic ion transport and metabolism
Q	180	3.9	Secondary metabolites biosynthesis, transport and catabolism
R	583	12.5	General function prediction only
S	414	8.9	Function unknown
-	1,554	27.1	Not in COGs

## Insights into the genome

### Plasmids and phages

Genome sequencing of *S. mucosus* DSM 16094^T^ resulted in 30 scaffolds. In the species description, it was reported that this strain contains at least seven plasmids (550, 467, 184, 140.8, 110.6, 98.2 and 30.8 kb) [7]. However, the identification of plasmids in the genome was difficult because typical replication modules comprising the characteristic replicase and the adjacent *parAB* partitioning operon are missing [41]. Nevertheless, comprehensive BLASTP searches with plasmid replicases from *Rhodobacterales* revealed the presence of three RepA and two RepB genes, whereas RepABC-type and DnaA-like replicases were absent from the genome sequence. General genomic features of the chromosome and four putative extrachromosomal replicons are listed in Table 5, whereas locus tags of the replicases and the large *virB4* and *virD4* genes of type IV secretion systems are presented in Table 6. The localization of the chromosomal replication initiator DnaA documents that (at least) scaffold 3 represents the chromosome. The 350 kb scaffold 5 contains a RepA-a type replicase [42] and a characteristic type IV secretion system (T4SS) comprising the relaxase VirD2 and the coupling protein VirD4 as well as the complete *virB* gene cluster for the transmembrane channel (Table 6 [43]). It probably represents a mobilizable extrachromosomal element. Scaffolds 18 and 28 contain RepA-b and RepB-I type replicases, respectively (Table 5), and may represent two additional plasmids of this species. However, the presence of plasmid replicases does not unequivocally correlate with extrachromosomal elements, as these genes may also reflect inactivated orphans or pseudogenes. Thus, the total number of *S. mucosus* plasmids cannot be exactly determined based on the draft genome sequence. A potential fourth plasmid is represented by the large 702 kb scaffold 2 that contains a RepA-c as well as a RepB-II replicase (salmuc_01514; salmuc_01780). However, the presence of typical CRISPRs representing the defense system against phage attacks [44] favors a chromosomal affiliation for scaffold 2.

**Table 5:** General genomic features of the chromosome and putative extrachromosomal replicons from *S. mucosus* DSM 16094^T†^

**Replicon**	**Scaffold**	**Replicase**	**Length** (bp)	**GC** (%)	**Topology**	**No. Genes**
Chromosome	3	DnaA	550,914	68	linear**	522
Plasmid 1	5	RepA-a	349,771	67	linear**	353
Plasmid 2*	18	RepA-b	45,096	59	linear**	42
Plasmid 3*	28	RepB-I	15,330	70	linear**	11
Plasmid 4*	2	RepA-c RepB-II	702,451	62	linear**	709

^†^ Number of genes deduced from automatic annotation.

*assignment uncertain

**circularity not experimentally validated.

**Table 6:** Integrated Microbial Genome (IMG) locus tags of *S. mucosus* DSM 16094^T^ genes for the initiation of replication and type IV secretion systems (T4SS) required for conjugation.

**Replicon**	**Replication initiation**	**Type IV Secretion**		
	Replicase	Locus Tag	VirB4	VirD4
Chromosome	DnaA	salmuc_02154	-	-
Plasmid 1	RepA-a	salmuc_03210	salmuc_02871	salmuc_03216
Plasmid 2*	RepA-b	salmuc_05477	-	-
Plasmid 3*	RepB-I	salmuc_05734	-	-
Plasmid 4*	RepA-c RepB-II	salmuc_01514 salmuc_01780	-	-

*assignment uncertain.

The genome sequence of *S. mucosus* DSM 16094^T^ reveals that this strain must encounter continuous attack by phages. Regions of genes related to prophages are found at several sites throughout the genome (e.g., salmuc_02795 - 02809 and salmuc_02619 - 02632). Several genes encoding cas proteins (salmuc_01330, salmuc_01331 and 01333) indicate that a CRISPR defense system is functional in this strain. The large number of phage-related genes integrated into the genome could mediate frequent rearrangements of the DNA structure in this strain. Furthermore, this could indicate a possible exchange of genes with other species attacked by similar phages.

### Morphological traits reflected in the genome

Analysis of the genome sequence of *S. mucosus* DSM 16094^T^ revealed the presence of a high number of genes associated with putative production and biosynthesis of exopolysaccharides (salmuc_00030, salmuc_00724, salmuc_01174, salmuc_01693, salmuc_02911, salmuc_3919, salmuc_04853 and salmuc_05511). This finding is in accord with a recent study by Llamas and colleagues, who characterized the exopolysaccharides produced by strain A3^T^ in detail [11]. Interestingly, genes putatively associated with cellulose synthesis (salmuc_02978 and salmuc_02979) were also found.

Surprisingly, many genes involved in flagellar motility (e.g., salmuc_02151 - salmuc_02191, salmuc_04184 - 04236) and chemotaxis (e.g., salmuc_03613 - 03617) were observed, although this strain was described as non-motile in the original species description [7].

Genes associated with the synthesis of polyhydroxyalkanoates as storage compound (e.g., salmuc_03738, salmuc_03739 and salmuc_05206) as well as phasin (salmuc_03343) were also found.

### Nutrient limitation

Many saline environments, e.g., the central oceans, are characterized by a limitation of the essential nutrients iron and phosphorous. *S. mucosus* seems to have installed several mechanisms to overcome growth limitation caused by depletion of both elements. Iron is mainly acquired by the synthesis of siderophores and transported into the cell in its chelated form. Genes encoding ABC transporters for siderophores of the hydroxamate type (salmuc_02461 - 02463 and salmuc_02667 - 02669), as well as for hemin-bound iron (salmuc_00710 - 00712) were found. To satisfy the need for phosphorous, strain DSM 16094^T^ is able to mobilize organic phosphonates as alternatives to phosphate, which is indicated by a continuous array of 22 genes (salmuc_00786 - 00807) involved in the uptake and utilization of phosphonates.

### Metabolic plasticity

In contrast to the published description of *S. mucosus* [7], which suggests a strictly aerobic and chemoheterotrophic metabolism, the genome reveals an astonishing metabolic versatility. Besides genes for the degradation of organic substrates, we also found genes encoding enzymes for the utilization of alternative electron donors enabling facultative lithotrophic growth: a Sox multienzyme complex encoded by the genes salmuc_00587 - 00597 could be utilized for the oxidation of thiosulfate to sulfate, while molecular hydrogen may be utilized as electron donor by a multimeric uptake hydrogenase of the [NiFe]-type (salmuc_04814 - 04830). A further potential substrate is carbon monoxide, which might be oxidized by an aerobic-type carbon monoxide dehydrogenase encoded by the genes salmuc_05576 - 05578. Additional genes encoding subunits of carbon monoxide dehydrogenase were found dispersed at several sites in the genome. The metabolic plasticity of this species is further reflected in a multiple branched electron transport chain. The cascade starts from a NADH dehydrogenase (salmuc_03065 - 03088) or succinate dehydrogenase complex (salmuc_00519 – 00521), where ubiquinone is reduced to ubiquinol. Electrons can either be transferred from ubiquinol via a terminal cytochrome *bd* ubiquinol oxidase (salmuc_05386 - 05387) directly to oxygen, or transferred to a cytochrome *bc*_1_ complex reducing cytochromes. Reduced cytochromes can then interact with terminal oxidases reducing oxygen. Genes for at least two different cytochrome *c* oxidases were detected, being either of a putative *caa*_3_- (salmuc_05284 - 05285) or *cbb*_3_-type (salmuc_00548 - 00551). The chemiosmotic gradient generated in the electron transport chain can be used for the synthesis of ATP by an ATP synthase complex of the FoF1 type (salmuc_01101 - 01110). According to the genome sequence there is also the possibility that nitrate could be used as alternative electron acceptor in the absence of oxygen. In addition to a periplasmic nitrate reductase of the Nap-type (salmuc_04127 - 04129) genes for a copper-containing dissimilatory nitrite reductase (salmuc_05547), a nitric oxide reductase (salmuc_05554 and salmuc_05555) and a nitrous oxide reductase (salmuc_04123) were detected, resulting in a complete pathway for denitrification of nitrate to molecular nitrogen.

Interestingly, the genome sequence of *S. mucosus* DSM 16094^T^ further revealed the presence of a high number of genes associated with putative photoautotrophy. Next to a photosynthesis gene cluster (salmuc_05125 - 05164) RuBisCO-associated genes (samuc_03532 - 03534) involved in the fixation of CO_2_ via the Calvin-Benson cycle (salmuc_03531 - 03539) were observed. The presence of such genes indicates a putative photoautotrophic growth under certain conditions. The genome of this strain also encodes a blue light-activated photosensor (BLUF, salmuc_00318) that may play a role in the light-dependent regulation of photosynthesis genes. It is tempting to speculate that a genetic inventory allowing photoautotrophy reflects an evolutionary position at the root of the *Roseobacter* clade. Several members of this lineage are known to be capable of an aerobic photoheterotrophic metabolism, whereas photoautotrophic growth has not been reported yet. By analogy, with the scenario proposed for the evolution of aerobic photoheterotrophic *Gammaproteobacteria* [45,46], representatives of the *Roseobacter* clade may have lost genes for CO_2_ fixation following adaptation to aerobic environments characterized by electron donor limitation, thereby preventing utilization of the Calvin-Benson cycle, which demands an abundant supply of reducing power and energy.

However, none of the novel metabolic traits, which are predicted based on the genome sequence, could be verified experimentally in our laboratory so far. One explanation may be that under unfavorable growth conditions, e.g. anaerobiosis, lysogenic phages become activated, so that growth does not become apparent.
